# Skin flap complications after cochlear implantations

**DOI:** 10.1007/s00405-016-4107-1

**Published:** 2016-05-31

**Authors:** Wojciech Gawęcki, Michał Karlik, Łukasz Borucki, Joanna Szyfter-Harris, Maciej Wróbel

**Affiliations:** 1 Department of Otolaryngology and Laryngological Oncology, Poznań University of Medical Sciences, Poznań, Poland; 2 Department of Phoniatrics and Audiology, Poznań University of Medical Sciences, Poznań, Poland; 3 Department of Dermatology, Poznań University of Medical Sciences, Poznań, Poland

**Keywords:** Skin flap complications, Cochlear implantation, Rotational two-layer flap, Explantation, Reimplantation

## Abstract

The objective of the study was to analyse and present the surgical management strategy for major skin flap complications (MSFC) after cochlear implantations. Patients fitted with a titanium-silicone-coated implant of the same kind, operated on between 1994 and 2013 with a standardised procedure (1076 medical charts) were analysed. Analysis aimed to identify and study individuals with skin problems related to the cochlear implant treatment, i.e. requiring surgical treatment in hospital defined as MSFC and focused on incidence, risk factors and treatment of MSFC. MSFC were diagnosed in 1.76 % of patients: 2.06 % of children and 1.35 % of adults, 2.43 % after implantation with a long “C”-shaped incision and 1.28 % after short retroauricular incision. Registered risk factors included head trauma, acute otitis media, poor hygiene in children, and general comorbidities in adults. The primary intervention was dependent on skin complication severity and included revision surgery with wound closure over an implant (52.6 %) and revision surgery with explantation (47.4 %). Revision surgery without explantation was successful in 40 % and the most effective approach was debridement with a two-layer rotational flap. Explantation led to ultimate wound healing in all cases. Major skin flap complications after cochlear implantation are rare, but their treatment is complex and difficult. Revision surgery with resection of infected tissue, formation of a rotational two-layer flap preceded and supplemented by intensive targeted antibiotic therapy can be effective and should be the first treatment option. Spontaneous implant explantation, abscess formation or unsuccessful primary treatment necessitate implant removal as the ultimate solution.

## Introduction

Cochlear implantation is a generally admitted surgical procedure for individuals with severe to profound sensorineural hearing loss who cannot benefit from conventional hearing aids. This solution has been routinely used since the 1970s and thousands of people (children and adults) have been successfully implanted. The benefits of this solution are without any doubts. However, cochlear implantation, like every surgical procedure, can cause some complications. They are usually related to the patients and only sometimes to surgery or to the implant [1].

Skin flap complications after cochlear implantation are associated with infection and inflammation of soft tissue covering the cochlear implant body. They can be divided into minor, which usually resolve spontaneously or can be managed by local/topical ambulatory treatment, and major, which require treatment in hospital involving surgical procedures and, often, explantation. The frequency of major complications is reported in 1.08–8.2 % of implanted patients [2–5] and cannot be reduced over the years of experience.

The aim of this study is to analyse the incidence and causative factors of major skin flap complications (MSFC) after cochlear implantations in our group of patients and to present our experience in different treatment options and its effectiveness.

## Materials and methods

The study was based on the retrospective analysis of medical charts of 1076 patients operated on with cochlear implants in our department between 1994 and 2013: 632 children (302 female and 330 male, aged 1–18 years with the mean of 5.39 and median of 3.5) and 444 adults (227 female and 217 male, aged 18.5–81.5 years with the mean of 46.54 and median of 47.5). All patients were fitted with the same type of cochlear implant (silicone-coated implant body—Cochlear Ltd., Australia), with the same surgical protocol and postsurgical follow-up (Table 1). The only modification over the years was the type of skin incision. A long “C”-shaped incision was performed in 452 implantations; since 2007 it was changed into a short linear retroauricular incision (624 implantations).

**Table 1:** Cochlear implantation surgical protocol and follow-up in our department

Surgery	
Skin incision	Long “C”-shaped (until 2007)
	Short post-auricular (since 2007)
Bone bed formation	Yes (always)
Fixation of an implant body	Yes (always)
Suturing	3 Layers (muscle, subcutaneous tissue, skin)
Final dressing	Ointment with antibiotic
	Sterile dressing material
	Bandage
Postsurgical follow-up	
Change of dressing	First at second day after surgery
	Then every day
	Last on 10th day after surgery
Antibiotic	4 days (first dose just before surgery)
Discharge from hospital	3 days after surgery
Suture removal	10 days after surgery in adults
	Not necessary in children—absorbable suture
Processor activation	30 (±5) days after surgery

Analysis aimed to identify individuals with MSFC defined as skin flap complications requiring in-hospital treatment with surgical intervention. Out of 1076 medical charts, 19 were selected due to reported MSFC. Patients underwent the defined protocol of complication treatment (Table 2).

**Table 2:** Protocol of major skin flap complication treatment in our department

Treatment phase	Sequence	Aim of the treatment	Details
Targeted antibiotic therapy	Introduction to the treatment	To reduce inflammation before surgery	The antibiotic used depended on bacteriological results and it was usually used orally in ambulatory treatment and then continued intravenously or changed to different intravenous delivery after admission to the hospital
Revision surgery	Primary surgical intervention	Elimination of infection and decontamination of implant site	Debridement of the wound, resection of infected tissue, topical antibiotic and antiseptic fluid
		Wound closure	Three options (techniques): 1) Suture 2) Rotational skin flap 3) Rotational flap composed of two layers: skin with subcutaneous tissue (external layer) and muscle with fascia (internal layer)
Explantation	Secondary surgical intervention and primary surgical intervention in selected cases: extensive soft tissue necrosis, spontaneous implant extrusion, abscess formation around the device	Elimination of the infection	Explantation of the device with surrounding infected soft tissue
		Prevention of cochlea fibrosis	Preservation of an implant electrode
Reimplantation		Hearing restoration	Three options: 1) Implantation of the contralateral ear before explantation 2) Implantation of the contralateral ear after explantation 3) Implantation of the same ear after explantation

A project database was created with a list of data to be collected: (a) incidence (frequency, onset and duration of complaints), (b) risk factors (age, sex, general medical condition, episodes of head trauma, hygiene, variation in surgical protocol, bacteriology) and (c) treatment (options and outcomes).

Obtained data were stored and statistically analysed in a MS Excel database. To compare the frequency of MSFC in the children versus adults group, in female versus male and in the long versus short incision group, a test of proportion was used. The investigation was approved by the local Ethics Committee.

## Results

### Incidence

**Fig. 1:**
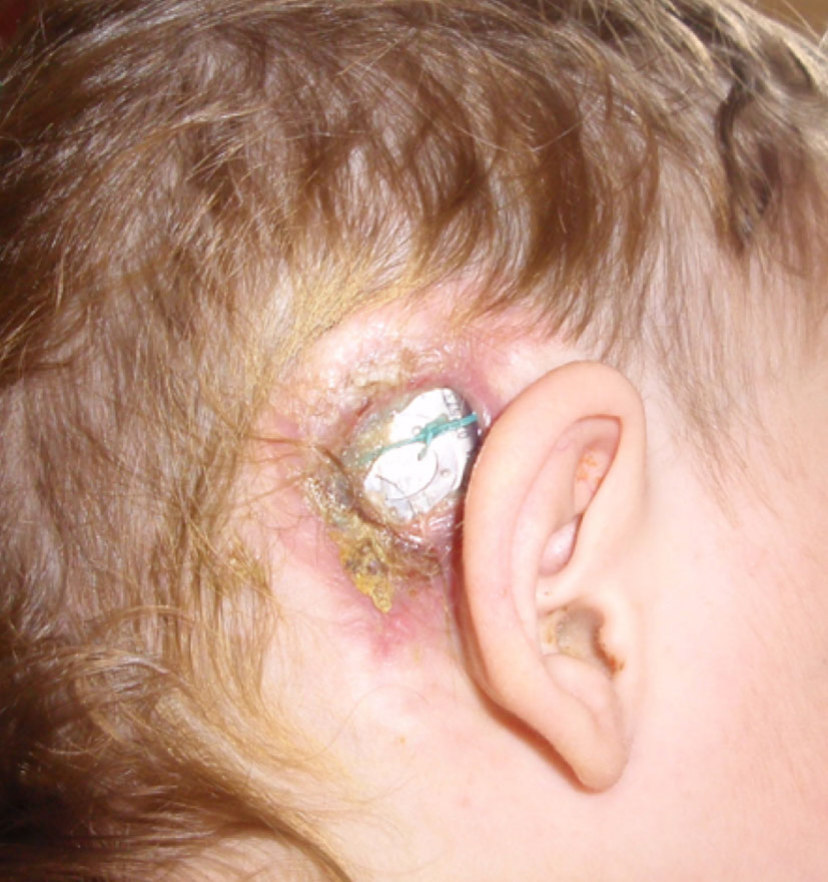
Major skin flap complication

**Fig. 2:**
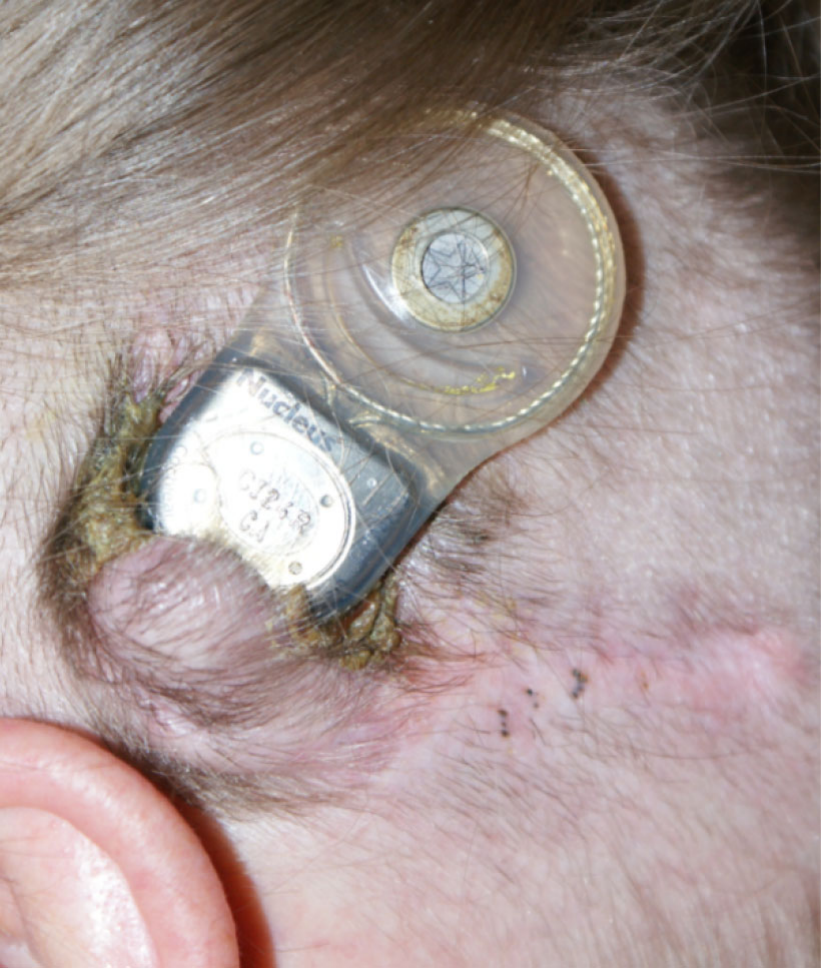
Major skin flap complication with spontaneous explantation of cochlear implant body

MSFC were detected and treated in 1.76 % (*n* = 19) out of a total 1076 implantations. In two cases, skin complications were detected after a second implantation (Table 3, patients 8 and 19). The onset of skin complication ranged from 1 month after primary implantation up to 10 years and 7 months afterwards, with a mean time of 33.2 months. The symptoms of MSFC comprised gradually developing redness, ulceration and soft tissue defect as well as the acute painful abscess of the operated side. Patients with MSFC are presented in Table 3 and examples of MSFC are presented in Figs. 1 and 2.

**Table 3:** Patients with major skin flap complications (according to the date of first symptoms)

Patient		Cochlear implantation					Skin flap complication							Second cochlear implantation	
							Date	Aetiology		Treatment					
No	Initials (sex)	Date	Age (years)	Implant type	Ear	Incision		Bacteriology	Additional risk factors	Antibiotic	Revision surgery	Revision surgery successful	Explantation	Ear	Date
1	PM (M)	Nov 2000	2.0	Cochlear CI 24 R(ST)	R	C	Apr 2003	*Staphylococcus aureus*	Acute otitis media (Feb and Jul 2001)	Sulfamethoxazole with trimethoprim, cefuroxime	Debridement and suture—Jun 2003	No	Feb 2005	L	Dec 2006
											Debridement and suture—Sept 2003	No			
											Debridement and suture—May 2004	No			
2	MM (M)	Nov 1999	43.0	Cochlear CI 24 M	L	C	Jun 2003	*Pseudomonas aeruginosa*	Chronic otitis media—open cavity After cardiac infarct	Ciprofloxacin, cefuroxime	Debridement and suture—Oct 2003	No	Jan 2006	L	Nov 2011
											Rotational skin flap—Nov 2004	No			
											Debridement and suture—Jun 2005	No			
3	SF (M)	Jul 2005	11.5	Cochlear CI 24 R(ST)	L	C	Sept 2005	No data	No	Cefuroxime, metronidazole	No (extensive soft tissue reaction)	–	Jan 2006	No data	–
4	DD (F)	May 2002	47.5	Cochlear CI 24 R(CS)	L	C	Oct 2005	No data	No	Clindamycin, metronidazole	Debridement and suture—Jan 2006	No	Aug 2006	R	Jun 2007
5	LMP (M)	Aug 2005	2.5	Cochlear CI 24 R(CA)	R	C	Jan 2006	No data	No	Cefuroxime	Debridement and suture—Jul 2006	No	No	No	–
											Rotational skin flap and free skin flap (skin from groin)—Nov 2006	Yes			
6	ŻN (F)	Jul 2007	1.5	Cochlear CI 24 RE(CA)	R	RA	Jul 2008	*Staphylococcus aureus*	Poor hygiene	Clindamycin	No (extensive soft tissue necrosis and poor hygiene)	–	Nov 2008	R	Jun 2009
7	JN (F)	Sept 2008	2.0	Cochlear CI 24 RE(CA)	R	RA	Jan 2009	No data	Smallpox infection	Clindamycin	No (abscess around implant body)	–	Mar 2009	L	Sept 2009
8	ŻN (F)	Jun 2009	3.5	Cochlear CI 24 RE(CA)	R	RA	Sept 2009	*Staphylococcus aureus*, *Streptococcus dys. equisimilis*	Poor hygiene Reimplantation of previously explanted ear	Sulfamethoxazole with trimethoprim, erythromycin	No (extensive soft tissue necrosis and poor hygiene)	–	Sept 2009	L	Oct 2014
9	IK (M)	Nov 2003	4.0	Cochlear CI 24 R(CA)	L	C	Jan 2010	*Staphylococcus aureus*	Trauma of the head Poor hygiene	Cefuroxime	No (extensive soft tissue necrosis and poor hygiene)	–	Jun 2010	R	Mar 2010
														L	Apr 2011
10	MB (F)	May 2005	20.5	Cochlear CI 24 R(CA)	L	C	May 2010	*Staphylococcus aureus*, *Staphylococcus epidermidis*	No	Cefuroxime, amoxicillin with clavulanic acid	Rotational skin flap—Jul 2010	No	Jan 2011	R	Oct 2010
11	LW (F)	Sept 2005	14.5	Cochlear CI 24 R(CA)	R	C	Jun 2010	*Klebsiella oxytoca*, *Pseudomonas stutzeri*, *Enterobacter cloacae*, *Enterococcus gallinarum*, *Staphylococcus aureus*	Trauma of the head	Gentamicin, ampicillin, amoxicillin with clavulanic acid	Rotational skin flap—Apr 2011	No	Jun 2011	L	Jun 2011
12	WM (F)	Oct 2006	1.5	Cochlear CI 24 RE(CA)	R	C	Jun 2010	*Enterobacter cloacae*, *Staphylococcus aureus*	Trauma of the head	Amoxicillin with clavulanic acid, sulfamethoxazole with trimethoprim, ceftriaxone, gentamicin	Debridement and suture—Oct 2010	No	Feb 2011	L	May 2010
13	ZW (F)	Dec 2006	2.0	Cochlear CI 24 RE(CA)	R	C	Feb 2011	No bacteria identified	Acute otitis media with retroauricular abscess (Feb 2011)	Amoxicillin with clavulanic acid, clarithromycin, metronidazole, clindamycin, sulfamethoxazole with trimethoprim	Rotational two- layer flap—Sept 2011	Yes	No	L	Apr 2011
14	SW (F)	Jan 2001	4.5	Cochlear CI 24 R(ST)	L	C	Sept 2011	*Staphylococcus epidermidis*, *Staphylococcus haemolyticus*	Acute otitis media (Apr 2011)	Clindamycin, vancomycin, gentamicin	Debridement and suture—Sept 2011	No	No	No	–
											Rotational two-layer flap—Mar 2012	Yes			
15	PR (F)	Sept 2008	46.0	Cochlear CI 24 RE(CA)	L	RA	Sept 2011	*Staphylococcus aureus*	Trauma of the head Renal failure (dialysis) Diabetes After cardiac infarct	Cefuroxime	No (partial spontaneous evacuation of implant body)	–	Nov 2011	R	Nov 2011
16	CI (M)	Oct 2011	1.5	Cochlear CI 24 RE(CA)	R	RA	Dec 2011	*Staphylococcus aureus*, *Staphylococcus epidermidis*, Coagulase-negative *Staphylococcus*	Sepsis in newborn Born as premature	Linezolid, erythromycin	Rotational two-layer flap—Jun 2012	Yes	No	No	–
17	DT (F)	Jul 2012	53.0	Cochlear CI 24 RE(CA)	R	RA	Nov 2012	*Staphylococcus warneri*, *Staphylococcus cohnii*, *Staphylococcus haemolyticus*	No	Vancomycin, amoxicillin with clavulanic acid, sulfamethoxazole with trimethoprim	Rotational two-layer flap—Dec 2012	No	Mar 2013	R	Nov 2014
18	WJ (M)	Nov 2012	1.0	Cochlear CI 24 RE(CA)	R	RA	Jan 2013	*Escherichia coli*, *Staphylococcus aureus*, Coagulase-negative *Staphylococcus*	Exudative otitis media—ventilation tube and antromastoidectomy (Jun 2012) Contact with patient with meningitis (Jun 2012)	Amoxicillin with clavulanic acid, metronidazole, cefuroxime, sulfamethoxazole with trimethoprim	No (extensive soft tissue necrosis)	–	Jun 2013	L	Sept 2013
19	PR (F)	Nov 2011	49.5	Cochlear CI 24 RE(CA)	R	RA	Feb 2013	*Staphylococcus aureus*	Renal failure (dialysis) Diabetes After cardiac infarct	Cefuroxime, amoxicillin with clavulanic acid	No (partial spontaneous evacuation of implant body)	–	Apr 2013	No	–

### Risk factors

The frequency of MSFC in the paediatric population was 2.06 % (*n* = 13) and in adults 1.35 % (*n* = 6). The difference between those two groups (0.71 %) is not statistically significant [0.71 % Chi^2^(1) = 0.757, *p* = 0.3844, 95 % CI −1.1260; 2.3800].

The analysis of sex showed MSFC in 2,32 % of female and 1,82 % of male in paediatric group [difference 0.4997 % Chi^2^(1) = 0.20, *p* = 0.6584, 95 % CI −2.0112; 3.1596, result statistically insignificant] and 2.2 % of female and 0.46 % of male in adults group [difference 1.7418 % Chi^2^(1) = 2.52, *p* = 0.1121, 95 % CI −0.8130; 1.7418, result statistically insignificant).

Additional patient-related risk factors were detected in 73.6 % (*n* = 14) out of 19 patients with MSFC. Within the paediatric group, the most common were acute otitis media (3 months to 10 years after implantation): 23.1 % (3 of 13), trauma of the head: 23.1 % (3 of 13) and poor hygiene: 23.1 % (3 of 13). In adults, general medical conditions, e.g. a history of cardiac infarction, diabetes or renal failure were observed in 50 % (3 of 6) cases.

The frequency of MSFC after cochlear implantation with long “C”-shaped incision was 2.43 % (11 of 452) and with short retroauricular incision was 1.28 % (8 of 624) [difference 1.15 % Chi^2^(1) = 2.00, *p* = 0.1573, 95 % CI −0.5749; 3.1636, result statistically insignificant].

Bacteriological culturing of MSFC showed *Staphylococcus aureus* to be the most frequent pathogen (78.6 %) followed by *Staphylococci* and Gram-negative spp.

### Treatment

Intensive targeted therapy with antibiotics was the first step of treatment in all patients (Table 1).

**Fig. 3:**
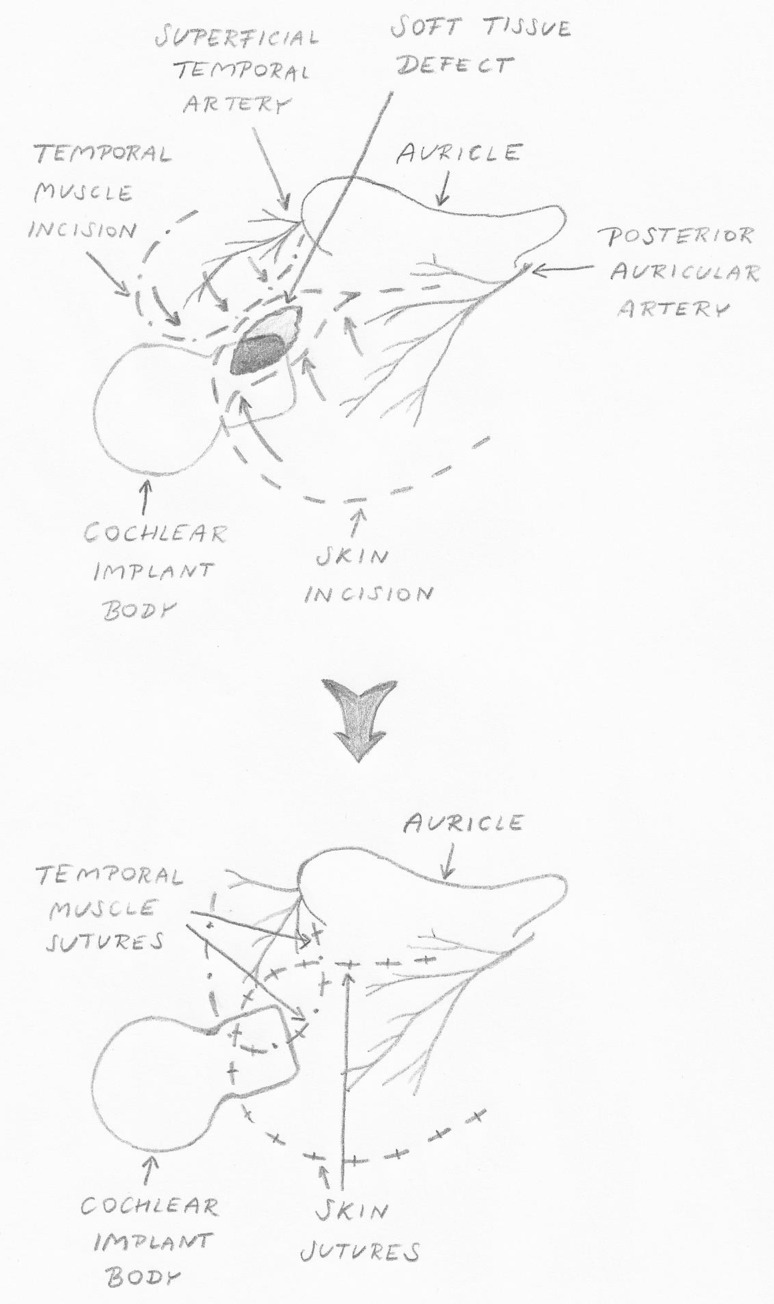
Rotational two-layer flap: the idea

**Fig. 4:**
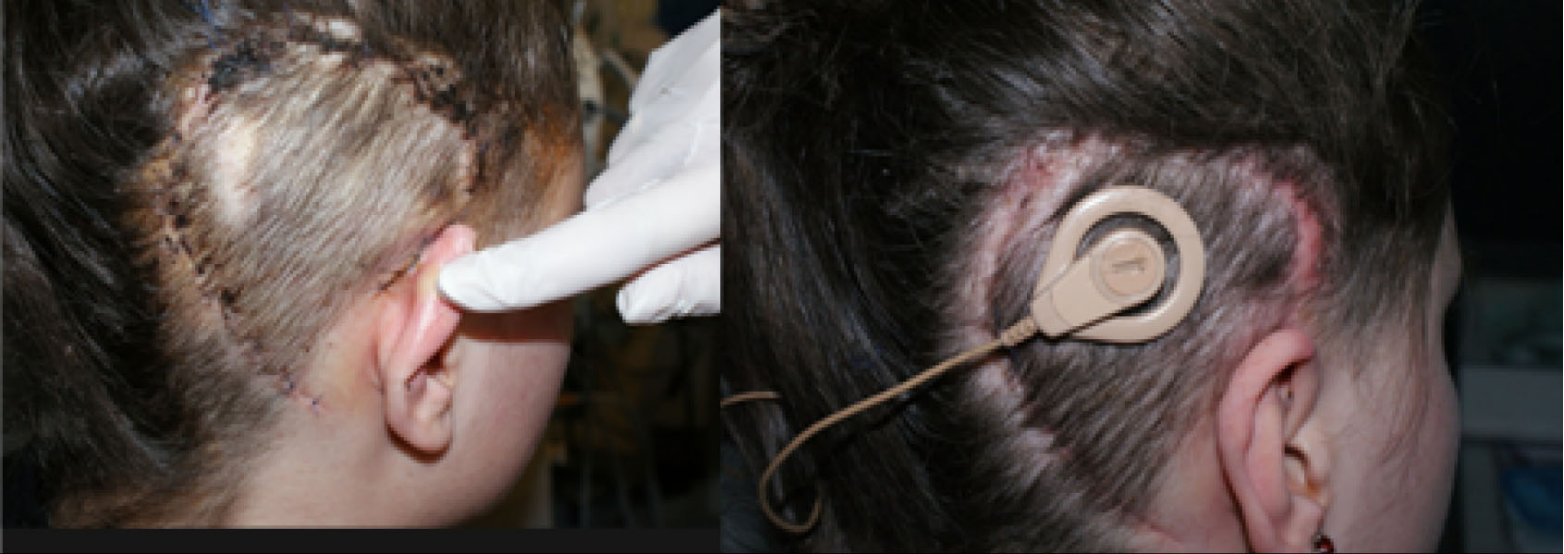
Patients after successful revision of skin flap complication with rotational two-layer flap

Revision surgery without explantation was done in 52.6 % (*n* = 10) of cases, and more than one operation was performed in 21.0 % (*n* = 4). Debridement and primary suturing of the wound was not effective. Rotational skin flap, performed in four cases, was not effective either except in one case supplemented by free skin flap reconstruction which gave a good and permanent positive result. Rotational two-layer flaps were done four times and were successful in three cases. The technique used since 2011 is based on preparation of two flaps: skin with subcutaneous tissue (external layer) and muscle with fascia (internal layer). The muscle was mobilised in the temporal region (superiorly to the pinna) and repositioned posteriorly and inferiorly, and skin was mobilised in the occipital region and rotated anteriorly to cover the targeted local complication. The idea of rotational two-layer flap is presented in Fig. 3. Patients after successful revision of MSFC by two-layer flap are presented in Fig. 4.

Explantation was performed in 78.9 % (*n* = 15) of cases: without previous revision in 47.4 % (*n* = 9) and after unsuccessful revision in 31.6 % (*n* = 6). Explantation led to complete wound healing in all patients.

Second cochlear implantation proceeded in 73.6 % (*n* = 14) of cases. Implantation of the contralateral ear before explantation of the affected one was performed in four cases and prior to revision surgery in one (patient 13). Implantation of the contralateral ear after explantation was performed in five cases (range 2 months to 5 years after). Reimplantation of the same ear was conducted in three cases (7 months to 5 years after explantation). In one patient (patient 9), the contralateral ear was implanted before and the affected ear was reimplanted 10 months after explantation, so this child uses two implants.

## Discussion

The frequency of MSFC after cochlear implantation reported in the literature ranges from 1.08 % [2] to 8.2 % [3]. The current study revealed skin complications in 1.76 % of the population, placing our patients within the same range. Such differences can be explained by the number of variables including factors related to patient, surgery or device.

The most frequent patient-related factors discussed in the literature are age and additional comorbidities. The influence of age on soft tissue infection is still an unsettled question. Younger age can be considered as a negative factor due to the higher risk of head trauma and immaturity of the immune system. Garcia-Valdecasas et al. [6] found no important differences between the frequency of MSFC in adults (5.7 %) and in children (6.2 %); however, the frequency in both groups was altogether high. Also, Low et al. [5] observed a similar frequency of MSFC in children (1.83 %) and adults (1.62 %). In our study, the frequency of MSFC in children was higher than in adults (2.06 versus 1.35 %) but these results are statistically insignificant. The influence of comorbidities is also not very clear. Hopfenspirger at al. [3] observed MSFC in a paediatric group more frequently in patients with specific chronic conditions, e.g. tracheotomy (23 %) in comparison to healthy implanted children (6.6 %). On the other hand, Garcia-Valdecasas et al. [6] observed no difference in the frequency of skin flap complications between patients with and without comorbidities. Allergic reactions to the device and radiotherapy of the temporal bone before cochlear implant surgery should also be taken into account [1, 5]. In our group, we have also noted additional local factors. In children, these were trauma of the head, which was noted in three cases (23.1 %), episodes of acute otitis media of the implanted ear (3 months to 10 years after implantation) in three cases (23.1 %) and poor hygiene in three cases (23.1 %). In the adult population, general comorbidities like history of cardiac infarction, diabetes and renal failure predominated. Additionally in adults group we have observed higher frequency of MSFC in female then in male (results statistically insignificant) which can be explain by the difference in soft tissue thickness covering the implant.

Ray et al. [7] showed that the rate of MSFC after cochlear implantation in patients operated on with a small incision (vertical post-aural incision) is significantly lower than in patients with large incision (retroauricular “C”-shaped incision or post-auricular incision with a horizontal posterior limb)—1.1 versus 2.3 %. Also, Davids et al. [2] emphasized that a small incision could be the reason for the small number of MSFC encountered in his paediatric cochlear implant group (1.08 %). Our study also showed that the change to a short incision could reduce the frequency of MSFC from 2.43 to 1.28 %.

Bacteriological examination is essential to ensure the use of proper and successful therapy with antibiotics supporting revision surgery. The literature reports the two most common pathogens as *Staphylococcus aureus* [3, 5, 8] and *Pseudomonas aeruginosa* [3, 6, 9]. In our material, swabs from the infected skin over the implant identified *Staphylococcus aureus* as the most frequent pathogen (78.6 %). *Pseudomonas aeruginosa* was found only in one patient.

The first surgical treatment option for MSFC is usually wound debridement, excision of infected tissue, decontamination of the device and skin closure. Rotational skin flap or dislocation of the transducer under healthy soft tissue can be performed. Such treatment should be supported by intensive targeted antibiotic therapy [10]. This management is aimed at eliminating inflammation and complete wound healing with the preserved device, but the success rate is variable and not warranted. The main reason for the treatment failure is the bacterial biofilm which covers the surface of an implant [11]. Low et al. [5] performed salvage surgery in six out of eight patients with MSFC with very good results. He used rotational skin flap in five cases, which was successful in two, and transposition of the device body in one case which was successful. Three out of five patients from the failed skin flap reconstruction group underwent subsequent transposition surgery with success. On the other hand, Garcia-Valdecasas et al. [6] tried conservative treatment and surgical cleaning in a group of nine cases with surgical-side infection, but it was not effective in all of them, so finally all these patients were explanted. Also, Hopfenspirger et al. [3] described 22 cases with MSFC after cochlear implantation of which 21 required explantations. In our department, revision surgery without explantation was always considered as a first treatment option but it was finally done in 52.6 %. Our surgical technique changed from debridement and suture of the wound, which failed in all cases, to wound debridement and covering the implant body by a rotational two-layer flap with a success rate of 75 %. This flap was composed of two layers: skin with subcutaneous tissue (external layer) and muscle with fascia (internal layer). Created flaps from healthy regions were subsequently superimposed onto each other over the exposed implant. The resultant closure with good peripheral blood supply was effective in 3 out of 4 cases with no further complication observed on follow-up visits.

The ultimate treatment of MSFC is implant explantation. It is usually indicated if primary revision surgery with device preservation has failed or it is advocated as an alternative to primary revision surgery if there is: (a) a high risk of intra-cranial complication; (b) a severe wound breakdown with complete extrusion of the cochlear implant body or (c) an allergic reaction to the device or foreign body reaction with device failure [1, 5]. In our patients with MSFC, explantation was performed in 78.9 % of cases (in 47.4 % without previous revision surgery and in 31.6 % after unsuccessful revision). Explantation always led to complete wound healing. Similar to others [2, 5, 6, 9], the electrode was cut and left inside the cochlea to prevent cochlea obliteration and to facilitate future reimplantation.

Second cochlear implantation is a very important issue for patients after explantation or qualified to explantation. Depending on the audiological conditions in the contralateral ear and soft tissue status after explantation, the contralateral or previously explanted ear can be implanted. The contralateral ear can be also implanted even before explantation to avoid a period without any device. In our study, second cochlear implantation was done in 73.6 % of cases (*n* = 14). In all but two cases requiring second explantation it was well tolerated.

## Conclusions

MSFC after cochlear implantations are rare, but their treatment is complex and difficult. Revision surgery with resection of infected tissue and formation of a rotational two-layer flap preceded and supplemented by intensive targeted antibiotic therapy can be effective and should be the first treatment option. Spontaneous implant explantation, abscess or unsuccessful primary treatment are indications for implant removal as the ultimate solution. Explantation, if possible, should be preceded or followed by cochlear implantation on the contralateral ear, or followed by reimplantation of the same ear after wound healing.
